# Big data analytics and machine learning in hematology: Transformative insights, applications and challenges

**DOI:** 10.1097/MD.0000000000041766

**Published:** 2025-03-07

**Authors:** Emmanuel Ifeanyi Obeagu, Christiana Uchenna Ezeanya, Fabian Chukwudi Ogenyi, Deborah Domini Ifu

**Affiliations:** a Department of Biomedical and Laboratory Science, Africa University, Mutare, Zimbabwe; b Department of Computer Science, National Open University of Nigeria, Abuja, Nigeria; c Department of Electrical, Telecommunication and Computer Engineering, Kampala International University, Kampala, Uganda.

**Keywords:** artificial intelligence, big data analytics, gene editing, genomics, hematology, machine learning

## Abstract

The integration of big data analytics and machine learning (ML) into hematology has ushered in a new era of precision medicine, offering transformative insights into disease management. By leveraging vast and diverse datasets, including genomic profiles, clinical laboratory results, and imaging data, these technologies enhance diagnostic accuracy, enable robust prognostic modeling, and support personalized therapeutic interventions. Advanced ML algorithms, such as neural networks and ensemble learning, facilitate the discovery of novel biomarkers and refine risk stratification for hematological disorders, including leukemias, lymphomas, and coagulopathies. Despite these advancements, significant challenges persist, particularly in the realms of data integration, algorithm validation, and ethical concerns. The heterogeneity of hematological datasets and the lack of standardized frameworks complicate their application, while the “black-box” nature of ML models raises issues of reliability and clinical trust. Moreover, safeguarding patient privacy in an era of data-driven medicine remains paramount, necessitating the development of secure and ethical analytical practices. Addressing these challenges is critical to ensuring equitable and effective implementation of these technologies. Collaborative efforts between hematologists, data scientists, and bioinformaticians are pivotal in translating these innovations into real-world clinical practice. Emphasis on developing explainable artificial intelligence models, integrating real-time analytics, and adopting federated learning approaches will further enhance the utility and adoption of these technologies. As big data analytics and ML continue to evolve, their potential to revolutionize hematology and improve patient outcomes remains immense.

## 1. Introduction

The field of hematology has witnessed a paradigm shift with the advent of big data analytics and machine learning (ML). These technologies leverage vast and complex datasets to derive actionable insights, fundamentally transforming approaches to diagnostics, prognostics, and therapeutic interventions. Hematology, with its reliance on intricate data from diverse sources such as genomic sequencing, clinical laboratory tests, and advanced imaging modalities, is uniquely positioned to benefit from these innovations. As the volume of available data grows exponentially, the ability to analyze and interpret it effectively becomes essential to advancing patient care.^[1]^ Big data analytics encompasses a set of methodologies designed to handle massive, high-dimensional datasets that are beyond the capacity of traditional data-processing techniques. In hematology, these datasets are derived from electronic health records (EHRs), high-throughput genomic data, proteomics, and imaging studies, among other sources. The integration of these datasets enables a comprehensive understanding of disease mechanisms, facilitates the discovery of novel biomarkers, and informs the development of targeted therapies. The power of big data lies not only in its size but also in its ability to reveal patterns and correlations that may otherwise go unnoticed.^[2]^ Machine learning, a subset of artificial intelligence, has further enhanced the analytical capabilities of big data. By using algorithms capable of learning from data and making predictions, ML has emerged as a powerful tool in hematology. Techniques such as supervised learning, unsupervised learning, and reinforcement learning are applied to classify diseases, predict patient outcomes, and optimize treatment plans. For instance, convolutional neural networks (CNNs) have shown remarkable success in analyzing peripheral blood smears and bone marrow aspirates for the detection of hematological malignancies.^[3]^

The integration of big data analytics and ML in hematology has already demonstrated its potential in various domains. For example, predictive analytics is increasingly being used to stratify patients into risk categories for diseases such as leukemia and thrombosis, enabling timely interventions. Similarly, unsupervised clustering algorithms are uncovering hidden subtypes of diseases, thereby refining diagnostic criteria and enhancing the personalization of therapies. These developments have laid the foundation for precision hematology, a field that aims to tailor medical care to the individual characteristics of each patient.^[4]^ The promise of these technologies lies in their ability to bridge the gap between bench and bedside, transforming basic research findings into clinical applications. This requires interdisciplinary collaboration between hematologists, data scientists, and bioinformaticians to develop robust algorithms and analytical frameworks. Moreover, regulatory bodies must establish guidelines to ensure that ML models are validated and meet safety and efficacy standards before their widespread implementation in clinical settings.^[5]^ Emerging trends in explainable artificial intelligence (XAI) and federated learning offer potential solutions to some of these challenges. XAI aims to make ML models more transparent and interpretable, thereby increasing their acceptance among clinicians. Federated learning, on the other hand, enables collaborative data analysis across institutions while preserving patient privacy, thus addressing issues related to data sharing and security.^[6]^

## 2. Aim

The aim of this review is to explore the transformative role of big data analytics and ML in the field of hematology.

## 3. Rationale

The rapidly growing complexity and volume of data in hematology, fueled by advances in genomics, proteomics, and imaging technologies, demand innovative analytical approaches for effective utilization. Traditional methods of data analysis are increasingly inadequate for managing these datasets and extracting clinically actionable insights. Big data analytics and ML have emerged as transformative tools capable of addressing these challenges by offering powerful solutions for pattern recognition, predictive modeling, and decision support. Hematological disorders, ranging from malignancies such as leukemia to inherited conditions like sickle cell anemia, often require intricate diagnostic and therapeutic approaches. Early detection, precise risk stratification, and personalized treatment are crucial for improving patient outcomes, yet these goals remain difficult to achieve using conventional techniques. Artificial intelligence (AI)-driven methodologies can bridge this gap by enabling more accurate diagnostics, refining treatment protocols, and identifying novel therapeutic targets. Despite their promise, the application of big data analytics and ML in hematology is still in its infancy, facing barriers such as data heterogeneity, lack of standardization, and concerns over model interpretability and ethical implications. This review is warranted to provide a comprehensive overview of how these advancements can be leveraged to revolutionize hematological practice, research, and patient care.

## 4. Review methodology

### 4.1. Literature search

A broad literature search was conducted using databases such as PubMed, Scopus, Web of Science, and Google Scholar. Keywords used in the search included:

- “big data analytics in hematology”
- “machine learning in hematology”
- “AI in blood disorders”
- “diagnostics in hematology using AI”
- “predictive analytics in hematology”
- “explainable AI in hematology”
- “federated learning in healthcare”

The search was limited to peer-reviewed articles published in English, with an emphasis on studies and reviews from the past decade to ensure relevance and inclusion of recent advancements.

### 4.2. Inclusion and exclusion criteria

- Inclusion criteria: Articles were included if they focused on the application of big data analytics or ML in hematology, highlighted advancements in diagnostics, therapeutics, or research, and addressed challenges or emerging solutions in this field.
- Exclusion criteria: Studies were excluded if they lacked specific focus on hematology, provided only theoretical discussions without practical implications, or were inaccessible due to paywalls.

### 4.3. Evolution of big data analytics in hematology

The evolution of big data analytics in hematology represents a significant milestone in the understanding and management of hematological disorders.^[7]^ Initially, hematology relied predominantly on manual observations, limited datasets, and traditional diagnostic methods.^[8]^ With the advent of computers and electronic data storage, the groundwork for data-driven approaches in hematology was laid.^[9]^ The digitization of patient records, advancements in diagnostic technologies, and the proliferation of genomic data repositories led to the accumulation of vast datasets in hematology.^[10]^ This influx of diverse data types laid the foundation for big data analytics. The deciphering of the human genome and subsequent advancements in genomic sequencing technologies triggered a revolution in hematology.^[11]^ Genomic profiling of hematological disorders became feasible, revealing intricate genetic alterations and disease-associated mutations.^[12]^ Beyond genomics, the integration of other “omics” data (such as transcriptomics, proteomics, and metabolomics) expanded the scope of understanding hematological disorders.^[13]^ This multi-omics approach provided a holistic view of disease biology. Data mining techniques and computational algorithms started gaining prominence, enabling the extraction of meaningful insights from large datasets.^[14]^ These approaches aided in pattern recognition, identifying disease signatures, and predicting treatment responses. The advent of ML and AI propelled hematology into a new era. ML algorithms enabled the development of predictive models, risk stratification tools, and diagnostic decision support systems, revolutionizing disease management.^[15]^

Digital imaging technologies facilitated the digitization of blood smears, bone marrow biopsies, and histopathological slides.^[16]^ Machine learning algorithms applied to digital pathology revolutionized diagnostic accuracy and automated disease classification.^[17]^ The integration of wearable devices and real-time monitoring tools contributed to continuous data collection.^[18]^ These technologies provided clinicians with dynamic patient data, enhancing personalized treatment strategies. Collaborative research initiatives, consortia, and data-sharing platforms emerged, fostering collaborative efforts among researchers, clinicians, and institutions. This sharing of data and knowledge accelerated research and improved data quality.^[19]^ With the burgeoning use of big data in hematology, ethical considerations, patient privacy, and regulatory frameworks became crucial.^[20]^ Guidelines and ethical standards were developed to govern the ethical use and protection of patient data.^[21]^ The evolution of big data analytics in hematology has been characterized by a shift from conventional diagnostic and treatment paradigms to data-driven, precision medicine approaches.^[22]^ As technologies continue to advance, the integration of big data analytics and AI-driven solutions is poised to redefine hematological research, diagnosis, and therapeutic interventions, promising more precise and personalized care for patients with hematological disorders.^[23]^

#### 4.3.1. AI in blood disorders

AI is significantly advancing the diagnosis, management, and understanding of various blood disorders. By leveraging ML and deep learning (DL) techniques, AI has proven to be a transformative tool in analyzing complex data sets, enhancing diagnostic accuracy, predicting outcomes, and personalizing treatments.

##### 4.3.1.1. AI in hematological malignancies

AI has shown remarkable potential in diagnosing and monitoring hematological cancers such as leukemia, lymphoma, and multiple myeloma. Machine learning models, including CNNs, have been utilized to analyze microscopic images of blood smears and bone marrow aspirates. These models can identify abnormal cell morphologies and classify subtypes of leukemia with accuracy comparable to, and sometimes exceeding, that of expert pathologists. Furthermore, predictive algorithms have been developed to assess patient-specific risks of progression or relapse, aiding in personalized treatment planning.^[8]^

##### 4.3.1.2. AI in hemoglobinopathies

Conditions such as sickle cell anemia and thalassemia have benefited from AI-powered tools designed to optimize disease management. AI algorithms are employed to detect abnormal hemoglobin patterns in electrophoresis data, enabling faster and more reliable diagnoses. In sickle cell anemia, AI models are being used to predict vaso-occlusive crisis episodes by analyzing biomarkers, clinical data, and patient-reported symptoms. Such predictive capabilities allow for timely intervention, reducing hospitalizations and improving patient outcomes.^[9]^

##### 4.3.1.3. AI in coagulation disorders

AI has revolutionized the management of bleeding and clotting disorders, including hemophilia and thrombophilia. Predictive models are used to assess bleeding risks, optimize clotting factor replacement therapy, and personalize treatment schedules. Deep learning algorithms are also being integrated into coagulation assays, improving the interpretation of test results and aiding in the diagnosis of conditions such as disseminated intravascular coagulation and antiphospholipid syndrome.^[10]^

##### 4.3.1.4. AI in bone marrow failure syndromes

In diseases like aplastic anemia and myelodysplastic syndromes, AI is enabling earlier detection through the analysis of genomic, transcriptomic, and proteomic data. AI algorithms can identify genetic mutations and epigenetic changes associated with these disorders, facilitating targeted therapy development. Additionally, predictive models are being used to assess the likelihood of disease progression to acute leukemia, supporting timely therapeutic interventions.^[11]^

##### 4.3.1.5. AI in rare blood disorders

For rare disorders such as paroxysmal nocturnal hemoglobinuria and hemophagocytic lymphohistiocytosis, AI offers new hope. By analyzing EHR data and laboratory results, AI models can identify patterns indicative of these conditions, leading to faster and more accurate diagnoses. These capabilities are especially valuable in settings with limited access to specialized expertise.^[12]^

#### 4.3.2. Diagnostics in hematology using AI

AI has revolutionized hematological diagnostics by enhancing accuracy, speed, and precision in detecting and classifying blood disorders. Through the integration of ML and DL techniques, AI-driven diagnostic tools are now capable of analyzing complex hematological data, offering clinicians invaluable insights and reducing the diagnostic burden.

##### 4.3.2.1. Automated blood smear analysis

Traditional blood smear examination requires significant expertise and time. AI-based systems, particularly CNNs, have been developed to automate this process. These systems can identify and classify different types of blood cells, including red blood cells, white blood cells, and platelets, as well as detect abnormalities such as anisocytosis, poikilocytosis, and inclusion bodies. For example, AI models can differentiate between subtypes of leukemia by identifying specific morphological changes in leukocytes with high accuracy.^[12]^

##### 4.3.2.2. Hematological malignancy diagnostics

AI is playing a pivotal role in diagnosing hematological cancers such as leukemia, lymphoma, and myeloma. By analyzing cytogenetic data, next-generation sequencing results, and flow cytometry outputs, ML algorithms can detect genetic mutations and chromosomal abnormalities indicative of these malignancies. Additionally, deep learning models have demonstrated the ability to classify leukemias into subtypes (e.g., acute myeloid leukemia [AML], ALL) based on bone marrow aspirates and peripheral blood smears, facilitating timely and accurate treatment decisions.^[13]^

##### 4.3.2.3. Coagulation disorders

The diagnosis of bleeding and clotting disorders, including hemophilia and thrombophilia, is greatly enhanced by AI. Advanced algorithms analyze coagulation profiles and laboratory test results (e.g., PT, aPTT, and D-dimer levels) to detect patterns associated with these conditions. For instance, AI-powered systems are being used to assess thrombotic risks in patients with antiphospholipid syndrome or disseminated intravascular coagulation, enabling early intervention.^[14]^

##### 4.3.2.4. Molecular diagnostics and genomics

AI excels in analyzing the vast and complex data generated by genomic and transcriptomic studies in hematology. Machine learning models can identify mutations, fusion genes, and expression patterns associated with blood disorders, such as mutations in the JAK2 or FLT3 genes linked to myeloproliferative neoplasms and AML, respectively. AI also facilitates the interpretation of multi-omics data, which integrates genomics, epigenomics, and proteomics, for a more comprehensive understanding of disease etiology and progression.^[15]^

##### 4.3.2.5. Rare and inherited blood disorders

AI has been instrumental in diagnosing rare and inherited blood disorders such as sickle cell anemia, thalassemia, and paroxysmal nocturnal hemoglobinuria. By analyzing patterns in hemoglobin electrophoresis data, flow cytometry results, and patient phenotypes, AI systems can provide rapid and accurate diagnoses. Moreover, predictive models can assess the likelihood of disease complications, such as vaso-occlusive crises in sickle cell anemia patients, allowing for preventive measures.^[16]^

##### 4.3.2.6. Point-of-care diagnostics

AI is also transforming point-of-care diagnostics in hematology by enabling portable devices to deliver reliable results in resource-limited settings. AI algorithms embedded in mobile apps or handheld devices analyze data from miniaturized sensors to diagnose anemia, malaria, or other hematological conditions with minimal infrastructure. This advancement is particularly beneficial for underserved regions, improving access to timely healthcare.^[17]^

### 4.4. Applications in disease understanding and diagnosis

The applications of big data analytics and ML in disease understanding and diagnosis within the field of hematology have been transformative, providing comprehensive insights and enhancing diagnostic precision.^[24]^ Big data analytics enable comprehensive genomic profiling, identifying genetic alterations, mutations, and aberrations associated with various hematological disorders.^[25]^ ML algorithms aid in deciphering complex genomic data, revealing disease-specific molecular signatures and subclassifications.^[26]^ ML-based algorithms assist in disease subclassification based on molecular profiles, enabling more accurate and refined categorization of hematological malignancies.^[27]^ This approach helps in distinguishing subtypes with varying prognoses and treatment responses. ML-driven decision support systems analyze patient data, including clinical parameters and genomic profiles, to aid clinicians in making accurate and timely diagnoses.^[28]^ These systems provide insights, flag potential abnormalities, and assist in differential diagnoses. Big data analytics combined with ML algorithms enhance the analysis of digital images from blood smears, bone marrow biopsies, and histopathological slides.^[29]^ Automated image analysis improves diagnostic accuracy, identifies morphological abnormalities, and assists in disease classification. ML models identify predictive biomarkers or genetic signatures associated with disease progression or treatment responses.^[30]^ These biomarkers aid in prognostication and treatment decision-making, guiding personalized therapeutic strategies. ML-based predictive models analyze patient data to identify individuals at higher risk of developing hematological disorders.^[31]^ This early risk assessment facilitates proactive interventions and personalized screening programs for at-risk populations.

ML algorithms predict drug responses and identify potential drug-resistant patterns in hematological disorders based on genomic and clinical data.^[32]^ This aids in selecting optimal therapies and mitigating treatment resistance. Big data analytics coupled with ML techniques enable precise monitoring of MRD levels post-treatment.^[33]^ ML algorithms predict disease relapse risk by analyzing MRD data, facilitating early intervention and treatment modification if needed.^[34]^ ML algorithms integrate diverse “omics” data (genomics, proteomics, transcriptomics) to generate comprehensive molecular profiles.^[35]^ This integrated approach enhances disease understanding by uncovering complex disease mechanisms and interactions. Continuous monitoring through wearable devices coupled with real-time data analysis assists in tracking hematological parameters and disease progression.^[36]^ ML models analyze streaming data, providing clinicians with dynamic patient information for real-time decision-making.^[37]^ These applications highlight how big data analytics and ML techniques are revolutionizing disease understanding and diagnostic capabilities in hematology, fostering more precise, data-driven, and personalized approaches to patient care.

### 4.5. Predictive models for treatment response and prognostication

Predictive models powered by big data analytics and ML are instrumental in predicting treatment responses and prognoses in hematological disorders.^[38]^ ML-based models analyze patient-specific data, including genomic profiles, clinical parameters, and treatment history, to forecast individual responses to specific therapies.^[15]^ These models identify patterns correlating with treatment efficacy, aiding clinicians in selecting the most effective treatments for individual patients. ML algorithms predict drug responses and anticipate potential resistance mechanisms by integrating genomic data with treatment outcomes.^[39]^ This allows for personalized treatment selection, avoiding ineffective therapies and anticipating the need for alternative strategies.

ML-driven prognostic models utilize patient data to stratify individuals into risk categories based on disease progression, survival outcomes, and recurrence probabilities.^[40]^ These models incorporate diverse data types to predict patient-specific prognoses, guiding treatment decisions and surveillance protocols. ML-based analyses of MRD data provide insights into disease persistence post-treatment.^[41]^ Predictive models use MRD measurements to forecast the risk of disease relapse, facilitating timely interventions or treatment modifications. ML algorithms continuously monitor treatment responses by analyzing evolving patient data.^[3]^ These models provide real-time feedback on treatment efficacy, allowing clinicians to adapt therapies based on changing disease dynamics. Predictive models guide the development of tailored therapeutic strategies by identifying patient-specific factors influencing treatment responses.^[42]^ This facilitates personalized treatment plans based on individual characteristics and molecular profiles. ML-driven predictive models support adaptive treatment protocols by analyzing ongoing treatment responses.^[43]^ These models enable clinicians to modify therapies, adjust dosages, or switch treatments based on real-time patient-specific data. ML algorithms integrate diverse datasets, including clinical records, genomic profiles, and treatment outcomes, to generate comprehensive predictive models.^[44]^ This integrated approach improves the accuracy and reliability of treatment response predictions. Continuous validation and refinement of predictive models against new datasets and evolving patient profiles ensure their accuracy and generalizability.^[45]^ Iterative improvements enhance the reliability of these models for clinical application. ML-based predictive models aid in designing more efficient and informative clinical trials.^[44]^ These models help identify patient subgroups likely to respond to investigational therapies, expediting drug development and precision medicine approaches. Predictive models fueled by big data analytics and ML techniques are transforming treatment decision-making in hematological disorders, paving the way for more personalized, effective, and adaptive therapies tailored to individual patient profiles.^[46]^ Continued research and validation are essential to refine these models and translate them into routine clinical practice.

## 5. ML models in hematology

ML models have shown significant promise in various fields of medicine, including hematology. They are used to assist in diagnostics, treatment prediction, patient risk stratification, and research advancements.^[47]^ ML models can distinguish between different types of blood cells, aiding in the diagnosis of conditions like leukemia, anemia, and other blood disorders by analyzing microscopic images of blood smears. Algorithms can assist in diagnosing and classifying different types and stages of blood cancers, such as leukemia, lymphoma, and myeloma, by analyzing genetic data, cell morphology, and other clinical parameters. ML models can help predict disease progression, relapse, or patient outcomes by analyzing various patient data, such as genetic information, laboratory results, imaging, and treatment history. They aid in predicting a patient’s response to specific therapies, optimizing treatment plans, and reducing adverse effects by analyzing historical treatment data and patient characteristics. ML is used in identifying potential drug candidates by analyzing molecular structures, interactions, and genomic data related to hematological disorders. ML models help in analyzing vast amounts of genomic data to identify genetic markers, mutations, and pathways associated with different blood disorders. ML algorithms streamline and automate laboratory processes, such as blood sample analysis, reducing human error and improving efficiency.^[47]^ ML-powered systems aid healthcare professionals in making better-informed decisions by analyzing patient data and suggesting potential diagnoses or treatment options. ML algorithms aid in analyzing complex flow cytometry data to characterize and classify blood cells. ML assists in interpreting vast genomic sequencing data to identify mutations or variations associated with hematological diseases. Development of predictive models to anticipate disease progression or response to specific treatments in conditions like anemia, hemophilia, or thrombosis. The integration of ML in hematology continues to evolve, showing great potential to improve diagnostics, treatment strategies, and patient care in blood-related disorders. However, continuous validation and refinement are crucial to ensure the accuracy and reliability of these models in clinical practice.

## 6. Big data techniques in hematology

Big data techniques in hematology involve the utilization of large volumes of diverse data, often including genomic, clinical, imaging, and experimental data, to derive insights, patterns, and correlations that can enhance our understanding of blood-related disorders. These techniques leverage advanced computational methods to analyze and interpret complex datasets in hematology.^[48]^ High-throughput sequencing generates vast amounts of genetic data. Big data techniques help in analyzing this data to identify mutations, genetic markers, and pathways associated with hematological disorders. Identifying genetic variations and mutations in large datasets to understand disease mechanisms and personalize treatment approaches. Extracting and analyzing structured and unstructured data from EHRs to identify patterns, risk factors, and treatment outcomes related to blood disorders. Analyzing data from a large population to identify trends, risk factors, and disease prevalence within specific demographics.^[48]^ Analyzing large collections of digital pathology images of blood smears or bone marrow samples to aid in disease diagnosis and classification. Applying big data analytics to radiological images (such as MRI, CT scans) to extract quantitative features and patterns for disease diagnosis and monitoring in hematology.

## 7. Machine learning integration

Predictive Analytics: Using machine learning models on large datasets to predict disease progression, treatment response, and patient outcomes based on various parameters.^[49]^

Clustering and Pattern Recognition: Identifying subtypes or clusters within hematological diseases to personalize treatment strategies or understand disease heterogeneity.^[49]^

Natural Language Processing (NLP): Analyzing text data from medical records, research papers, or literature to extract valuable insights and facilitate knowledge discovery in hematology.^[49]^

Big data techniques play a crucial role in advancing hematology research, diagnosis, treatment, and patient care. They enable the extraction of meaningful insights from vast and diverse datasets, leading to improved understanding, more precise diagnostics, and better-tailored treatments for blood-related disorders.^[49]^

## 8. The emerging AI in hematology

AI is revolutionizing hematology, offering innovative solutions for diagnostics, treatment, and research. AI technologies, including ML, DL, and NLP, are designed to process vast amounts of data and extract meaningful insights. These capabilities are particularly impactful in hematology, where diverse datasets – ranging from blood cell morphology to genomic data – demand sophisticated analytical tools. AI is poised to address challenges in disease detection, risk assessment, and personalized treatment, fostering a new era of precision medicine in hematology.^[7]^ One of the most notable applications of AI in hematology is its role in disease diagnostics. AI-powered tools, such as CNNs, are increasingly employed for analyzing blood smears and bone marrow aspirates. These models can detect and classify hematological disorders like leukemia, lymphoma, and anemia with accuracy comparable to expert pathologists. Moreover, AI algorithms have demonstrated the ability to identify subtle morphological changes in blood cells, enabling early diagnosis and monitoring of conditions like myelodysplastic syndromes and sickle cell disease.^[8]^

AI is also driving advancements in prognostic modeling and risk stratification. By analyzing patient data, including clinical histories, laboratory results, and genetic profiles, predictive models can forecast disease progression and treatment responses. For example, AI algorithms are used to identify high-risk patients with AML, guiding treatment decisions and improving outcomes. In addition, ML-based tools are enhancing the precision of bone marrow transplant matching, optimizing donor-recipient compatibility to reduce complications.^[9]^ Another frontier for AI in hematology is drug discovery and therapeutic optimization. AI-driven platforms are being utilized to identify novel drug targets and predict therapeutic efficacy. Reinforcement learning algorithms are aiding in the design of personalized treatment regimens, ensuring optimal drug dosages and minimizing adverse effects. In diseases like hemophilia and beta-thalassemia, AI is facilitating the development of gene therapies by analyzing complex genomic datasets and predicting gene-editing outcomes.^[10]^ Beyond diagnostics and therapy, AI is transforming hematology research by accelerating data integration and hypothesis generation. AI tools enable the integration of multi-omics data, including genomics, transcriptomics, and proteomics, to uncover novel biomarkers and pathways. NLP algorithms are mining scientific literature and EHRs to identify trends and correlations, fostering a deeper understanding of hematological disorders. These insights are catalyzing the development of targeted therapies and enhancing disease management strategies.^[11]^

Despite its promise, the adoption of AI in hematology faces several challenges. The “black-box” nature of many AI models raises concerns about interpretability and trustworthiness. Ensuring the quality and diversity of training datasets is critical to avoid biases that could compromise patient care. Moreover, the integration of AI into clinical workflows requires robust validation, regulatory approval, and clinician training. Addressing these challenges is essential to fully harness the transformative potential of AI in hematology.^[12]^ Emerging trends in XAI and federated learning offer solutions to some of these hurdles. XAI focuses on making AI models more transparent, enabling clinicians to understand and trust the decision-making process. Federated learning, which allows decentralized analysis of data across institutions, ensures patient privacy while facilitating collaborative research. These innovations, coupled with advancements in computational power and data science, are paving the way for the seamless integration of AI into hematology.^[13]^

### 8.1. Challenges and limitations

In the realm of employing big data analytics and ML in hematological disorders, several challenges and limitations persist, hindering their seamless integration and optimal utilization.^[50]^ Ensuring the quality, completeness, and harmonization of diverse datasets (genomic, clinical, imaging) poses a significant challenge. Inconsistent or fragmented data, data silos, and difficulties in integrating disparate data sources can affect the accuracy and reliability of predictive models. ML models often operate as “black boxes,” lacking interpretability and explainability in their decision-making processes.^[45]^ The inability to elucidate how models arrive at conclusions or treatment recommendations may limit clinician trust and acceptance.

The use of sensitive patient data for predictive analytics raises ethical concerns regarding patient privacy, consent, and data security.^[51]^ Strict adherence to ethical guidelines and regulatory frameworks is crucial to protect patient confidentiality and ensure data security. Biases inherent in training data or underrepresentation of certain populations may lead to biased predictions or models that do not generalize well to diverse patient populations.^[52]^ Addressing biases in datasets and ensuring model fairness is essential for equitable healthcare. Integrating predictive analytics into existing clinical workflows and EHR systems poses challenges.^[53]^ Resistance to change, lack of interoperability, and additional time demands on clinicians for model interpretation hinder seamless integration. Access to computational resources, expertise in AI/ML development, and data science skills may be limited in some healthcare settings.^[54]^ The need for specialized knowledge in advanced analytics creates barriers to implementation. The evolving landscape of regulations and standards related to AI in healthcare presents challenges. Ensuring compliance with regulatory frameworks, navigating legal complexities, and addressing liability concerns are critical.

Validating predictive models across diverse patient cohorts and clinical settings is essential for their reliability.^[55]^ Transparency in model development, validation, and performance metrics is necessary for gaining clinician and patient trust. Implementing and maintaining AI-driven models and big data analytics solutions may be cost-prohibitive for some healthcare institutions.^[56]^ Assessing the cost-effectiveness and long-term sustainability of these technologies is vital. ML models require continuous updates and refinement to adapt to evolving data and patient characteristics.^[45]^ The need for ongoing improvement and validation adds complexity to their implementation.

### 8.2. Integration into clinical practice

Integrating big data analytics and ML into clinical practice for managing hematological disorders involves various considerations and steps.^[57]^ Providing clinicians with training and education on the use of data analytics and ML in hematology is essential. Continuing education programs, workshops, and specialized training sessions help in fostering familiarity and competence in utilizing these technologies. Collaboration among hematologists, data scientists, bioinformaticians, and technology experts is vital for successful integration.^[58]^ Multidisciplinary teams enable the interpretation of complex data and the development of clinically relevant applications. Developing user-friendly interfaces and decision support tools that integrate seamlessly into existing EHR systems facilitates clinician engagement.^[59]^ These tools aid in interpreting data and providing actionable insights at the point of care. Validating predictive models and algorithms in real-world clinical settings is crucial for gaining clinician confidence.^[60]^ Robust evidence demonstrating the clinical utility and accuracy of these models is essential for widespread adoption. Educating patients about the role of data analytics and ML in their care fosters transparency and trust.^[61]^ Ensuring patient understanding and obtaining informed consent regarding the use of predictive models and data-driven approaches is imperative.

Adhering to regulatory standards and ethical guidelines regarding patient data privacy, consent, and confidentiality is essential.^[62]^ Compliance with HIPAA and other data protection laws is crucial for ethical implementation.^[63]^ Involving clinicians in the development and validation of predictive models fosters acceptance and adoption. Gathering feedback from clinicians helps in refining models to better suit clinical needs and workflows. Initiating pilot programs within healthcare institutions allows for iterative testing, refinement, and optimization of data-driven tools.^[64]^ Gradual implementation strategies mitigate disruption to clinical workflows. Ensuring ongoing maintenance, updates, and refinement of predictive models based on new data and feedback is vital. Establishing protocols for continuous improvement ensures the relevance and accuracy of these tools. Providing evidence of improved patient outcomes, enhanced diagnostic accuracy, or optimized treatment decisions through data analytics and ML tools is crucial for demonstrating their clinical value and justifying their integration.^[65]^

### 8.3. Future directions and emerging innovations

Looking ahead, several future directions and emerging innovations are poised to shape the landscape of big data analytics and ML in the management of hematological disorders.^[66]^ Advancements in integrating diverse “omics” data (genomics, transcriptomics, proteomics, metabolomics) will provide a more holistic understanding of hematological diseases.^[13]^ Multi-omics approaches will uncover complex disease mechanisms and potential therapeutic targets. Further development of single-cell technologies will enable the characterization of cellular heterogeneity within hematological malignancies.^[67]^ This granular insight will elucidate clonal evolution, identify rare cell populations, and inform personalized treatment strategies. Integrating genomics with immune profiling will advance immunotherapeutic strategies in hematological disorders.^[68]^ Understanding the tumor microenvironment and immune responses will guide the development of more effective immunotherapies.

Focus on developing ML models that are interpretable and explainable will enhance clinician trust and adoption.^[45]^ Explainable AI will provide transparent insights into model predictions and aid in understanding complex algorithms. Further integration of wearable devices and real-time monitoring tools will enable continuous data collection. ML-driven analytics of real-time patient data will facilitate dynamic treatment adjustments and remote patient monitoring. Continued advancements in ML algorithms for digital pathology and imaging analysis will improve diagnostic accuracy.^[46]^ Automated analysis of blood smears, bone marrow biopsies, and histopathological images will aid in disease classification. Research into gene editing technologies, such as CRISPR/Cas9, will pave the way for precise and personalized gene therapies for hematological disorders.^[69]^ Enhanced global collaborations and data-sharing initiatives will foster the creation of larger and more diverse datasets.^[70]^ Shared datasets will enable more robust analyses and discoveries. Further development of ethical guidelines and regulatory frameworks will ensure responsible and ethical use of AI in hematology.^[71]^ Emphasis on patient privacy, data security, and ethical AI practices will guide future implementations. Innovative trial designs using predictive analytics and AI-driven patient stratification will accelerate the development of targeted therapies.^[72]^ Adaptive trials will optimize treatment strategies based on patient-specific characteristics.

The narrative review is summarized in Table 1 showing the Summary of Big Data Analytics and Machine Learning in Hematology: Transformative Insights, Applications, and Challenges (provided by the authors).

**Table 1:** A summary of big data analytics and machine learning in hematology: transformative insights, applications, and challenges

Section	Key points
Introduction	- Overview of big data analytics and machine learning (ML) in hematology. - Their transformative potential for diagnostics, treatment, and research. - Challenges like data integration, quality, and ethical concerns.
Emerging AI in hematology	- AI technologies, such as machine learning and deep learning, are being integrated into hematological diagnostics and treatments. - AI enhances the accuracy and speed of detecting blood disorders like leukemia and anemia.
Applications of AI in blood disorders	- AI used for diagnosing hematological malignancies, such as leukemia, lymphoma, and multiple myeloma. - AI also assists in rare blood disorder diagnostics like sickle cell anemia and hemophilia. - Personalized treatment decisions are aided by AI-driven predictive models.
AI in hematology diagnostics	- AI-based automated blood smear analysis with CNNs improves the classification of blood cells. - Molecular diagnostics and genomics analyzed by AI assist in the detection of mutations and gene fusions associated with blood cancers.
AI in coagulation disorders	- AI helps diagnose bleeding and clotting disorders like hemophilia and thrombophilia. - Models predict thrombotic risks and optimize treatment plans for disorders such as DIC and antiphospholipid syndrome.
Challenges in AI implementation	- Data quality issues and model interpretability remain significant obstacles. - Ethical concerns regarding AI use, patient privacy, and data bias. - Integration of AI into clinical workflows requires standardization.
Future directions	- Advances in explainable AI (XAI), federated learning, and real-time analytics will improve model transparency and patient privacy. - Automated, AI-driven diagnostic workflows will enhance clinical efficiency and outcomes.
Conclusion	- AI is revolutionizing hematological diagnostics and treatments. - Continued research and overcoming challenges will maximize AI’s potential to improve patient care and outcomes in hematology.

AI = artificial intelligence, CNNs = convolutional neural networks, DIC = disseminated intravascular coagulation.

## 9. Conclusion

The integration of big data analytics and AI, particularly ML, has set the stage for a transformative era in hematology. These technologies offer unprecedented capabilities in disease diagnosis, prognostic modeling, and personalized treatment, addressing some of the most complex challenges in the field. From analyzing intricate blood cell morphologies to uncovering novel biomarkers through multi-omics integration, AI is significantly enhancing precision medicine. Furthermore, its applications in drug discovery and optimization of therapeutic regimens underscore its potential to improve patient outcomes and reshape clinical workflows.

However, the path to widespread adoption is not without obstacles. Technical challenges such as data heterogeneity, algorithm validation, and integration into clinical systems must be addressed. Ethical considerations, including bias in training data, patient privacy, and the transparency of AI models, are equally critical to ensuring equitable and trustworthy implementations. Collaborative efforts among hematologists, data scientists, and regulatory bodies will be vital in overcoming these barriers and translating AI innovations into practice. As advancements in explainable AI, federated learning, and computational capabilities continue, the future of AI in hematology appears bright. These technologies promise to bridge gaps in research and clinical care, fostering a new era of precision, efficiency, and innovation. By embracing these advancements and addressing the associated challenges, hematology can harness the full potential of AI to improve patient care and advance the field to new horizons.

## Author contributions

**Conceptualization:** Emmanuel Ifeanyi Obeagu.

**Methodology:** Emmanuel Ifeanyi Obeagu, Deborah Domini Ifu.

**Supervision:** Emmanuel Ifeanyi Obeagu.

**Validation:** Emmanuel Ifeanyi Obeagu.

**Visualization:** Emmanuel Ifeanyi Obeagu, Deborah Domini Ifu.

**Writing – original draft:** Emmanuel Ifeanyi Obeagu, Christiana Uchenna Ezeanya, Fabian Chukwudi Ogenyi, Deborah Domini Ifu.

**Writing – review & editing:** Emmanuel Ifeanyi Obeagu, Christiana Uchenna Ezeanya, Fabian Chukwudi Ogenyi, Deborah Domini Ifu.
